# 102例40岁以下青年非小细胞肺癌患者的临床特征及预后分析

**DOI:** 10.3779/j.issn.1009-3419.2013.02.03

**Published:** 2013-02-20

**Authors:** 莉莉 曲, 海峰 秦, 晓晴 刘, 红军 高, 俭杰 李, 伟霞 王, 传昊 汤, 万峰 郭, 晓燕 李

**Affiliations:** 100071 北京，军事医学科学院附属307医院肺部肿瘤内科 Department of Lung Cancer, Affiliated Hospital of Academy of Military Medical Science, Beijing 100071, China

**Keywords:** 肺肿瘤, 青年, 病理特征, 治疗, 预后, Lung neoplasms, Young, Pathological characteristic, Therapy, Prognosis

## Abstract

**背景与目的:**

青年（年龄≤40岁）非小细胞肺癌（non-small cell lung cancer, NSCLC）发病率呈上升趋势。本研究旨在分析青年NSCLC的临床病理生理特征、治疗及预后情况。

**方法:**

对102例资料完整的青年NSCLC患者进行回顾性分析。

**结果:**

女性所占比例为43.1%，男女比例为1.32:1；29.4%有吸烟史；以腺癌为主，占77.5%；以低分化癌为主，占64.1%；晚期肺癌（Ⅲb期及Ⅳ期）占87.8%。6例接受手术治疗患者的中位复发时间为13.5个月。87例接受一线化疗患者的客观有效率（objective response rate, ORR）为46.0%，疾控率（disease controled rate, DCR）为79.3%，中位肿瘤进展时间（time to progress, TTP）为5.0个月。38例接受表皮生长因子受体酪氨酸激酶抑制剂（epidermal growth factor receptor-tyrosine kinase inhibitors, EGFR-TKI）治疗患者ORR为40%，DCR为65.7%，中位TTP为5.5个月；12例二次或多次TKI治疗患者DCR为66.7%，中位TTP为3.0个月。

**结论:**

青年NSCLC中位确诊时间长，女性所占比例相对较高，与吸烟的相关性较弱，以分化差的晚期腺癌为主，确诊后应给予积极的综合治疗，但总体预后不佳。

## Abstract (en)

**Background and objective:**

The incidence of young non-small cell lung cancer (NSCLC) annually increases. The aim of this study is to analyze the clinical pathological characteristics of young (less than 40 years old) NSCLC patients.

**Methods:**

The data of 102 young NSCLC were retrospectively analyzed.

**Results:**

Among the 102 patients, 43.1% were women and 29.4% were smokers. The male-to-female ratio was 1.32:1. The most frequent histologic type was adenocarcinoma (77.5%). Tumor differentiation was mostly poor (64.1%), and 87.8% had stages Ⅲb and Ⅳ diseases. The median recurrence time of 6 patients who had tumor resection was 13.5 months. The objective response rate (ORR) of 87 patients who received first-line chemotherapy was 46.0%, the disease control rate (DCR) was 79.3%, and the median time to progression (TTP) was 5.0 months. The ORR of 38 patients who received epidermal growth factor receptor-tyrosine kinase inhibitors (EGFR-TKI) therapy was 40.0%, with a DCR of 65.7% and a median TTP of 5.5 months. The DCR of 12 patients who received EGFR-TKI twice or more times was 66.7%, with a median TTP of 3.0 months.

**Conclusion:**

The time from the first presenting symptom until diagnosis was usually long. The female proportion presented an upward trend and the correlation became attenuated between young NSCLC patients and smoking. Most of the young NSCLC patients had adenocarcinoma and poor tumor differentiation. Multidisciplinary and systematic therapies were needed to improve the poor prognosis of the young NSCLC patients.

肺癌发病率及死亡率逐年升高，现已成为恶性肿瘤中致命的头号杀手，其中尤以非小细胞肺癌（non-small cell lung cancer, NSCLC）为甚，其发病率约占肺癌总发病率的80%，且确诊时多为中晚期。NSCLC多发于40岁以上人群，其发病率随年龄的增长而逐渐升高，NSCLC确诊的中位年龄为71岁，男性75岁-79岁达发病高峰，女性为70岁-74岁^[1, 2]^。青年NSCLC（年龄≤40岁）相对少见，来自欧洲及日本的研究数据显示，青年NSCLC发病率呈逐年上升趋势^[3, 4]^，且其有与老年NSCLC不同的临床特点^[5]^。本研究收集307医院肺部肿瘤内科近10年收治的青年NSCLC患者的临床资料，回顾性分析其临床病理特征、治疗及预后情况，以期对青年NSCLC有初步认识。

## 1 资料与方法

### 1.1 病历资料

收集我科2001年1月-2011年10月收治的102例青年NSCLC患者（年龄≤40岁）临床资料。所有患者均经纤维支气管镜下刷检和（或）病理活检、转移淋巴结穿刺活检等确诊为NSCLC，肿瘤分期根据1997年肺癌国际TNM分期标准（2009年后采用2009版UICC新分期标准）。

### 1.2 研究方法

回顾性分析患者的性别、吸烟史、美国东部肿瘤协作组制定的体力状况评分（Eastern Cooperation Oncology Group performance status, ECOG PS）、体重下降情况、发病症状、出现临床症状至接受诊断治疗的时间、合并症、病理类型、临床分期、治疗及生存情况。

### 1.3 统计学方法

采用SPSS 17.0统计分析软件进行数据处理。生存期计算自确诊时间开始至死亡时间或末次随访日期（2012年4月）。

## 2 结果

### 2.1 一般资料

见表 1。102例患者中男性58例（56.9%），女性44例（43.1%），男女性别比为1.32:1，中位年龄36岁（19岁-40岁），其中年龄≤30岁的患者10例，占9.8%（10/102）。29.4%（30/102）患者有吸烟史（吸烟指数＞400），均为男性，女性患者中有1例有长期被动吸烟史。确诊时ECOG PS评分为0分-1分的患者占88.2%（90/102）。发病时3个月内体重较前下降≥5%者占15.7%（16/102）。有合并症的患者占8.8%（9/102），分别为肺结核3例、慢性乙型肝炎3例、脑垂体瘤1例、十二指肠溃疡1例及慢性结肠炎1例。7例患者其父亲或母亲有肺癌病史（6.9%, 7/102）。

**1:** 102例青年非小细胞肺癌患者一般特征 Characteristics of 102 young non-small cell lung cancer (NSCLC) patients

Characteristic	*n*(%)
Gender	
Male	58 (56.9)
Female	44 (43.1)
Smoking status	
Former and current	30 (29.4)
Never	72 (70.6)
ECOG PS status	
0-1	90 (88.2)
≥2	12 (11.8)
Weight loss	
≥5%	16 (15.7)
< 5%	86 (84.3)
Complication	
With complication	9 (8.8)
Without complication	93 (91.2)
Presenting symptoms	
Cough	34 (33.3)
Pain	22 (21.6)
Nausea	11 (10.8)
Superficial tumor	9 (8.8)
Fever	7 (6.9)
Physical examination	7 (6.9)
Trachyphonia	4 (3.9)
ECOG PS: Eastern Cooperation Oncology Group performance status.	

患者初始发病症状以咳嗽及骨痛最为多见，肺外全身症状以骨痛（21.6%, 22/102）、转移淋巴结或皮下转移结节（8.8%, 9/102）及发热（6.9%, 7/102）为主，6.9%患者因体检发现肺内占位而就诊，发病时同时有2个或以上症状的患者占30.3%（31/102）。患者自出现初始临床症状至确诊时间为7天-52个月，中位确诊时间为2.5个月。20例患者首次就诊时被误诊，延误治疗时间为0.5个月-12个月，主要误诊原因为肺炎或支气管炎（10例）、肺结核或结核性胸膜炎（7例）、恶性淋巴瘤（1例）、软骨炎（1例）、中耳炎（1例）。

### 2.2 肿瘤病理类型及分化程度

102例患者均有明确的病理诊断，其中腺癌占77.5（79/102），鳞癌占16.7%（17/102），大细胞癌及腺鳞混合癌各占2.9%（3/102）。79例腺癌中有48例原发灶为外周型，31例为中央型；而17例鳞癌的原发灶均为中央型。39例肿瘤分化程度明确，其中低分化25例（64.1%, 25/39），中低分化6例，中分化4例，高中分化1例，高分化3例。分化差的肿瘤（低分化及中低分化）占79.5%（31/39）。在有吸烟史的30例男性患者中有16例为鳞癌（53.3%, 16/30），13例为腺癌（43.3%, 13/30），1例为大细胞癌（3.3%, 1/30）。

### 2.3 肿瘤临床分期

98例肿瘤分期明确，其中Ⅰ期3例，Ⅱ期3例，Ⅲa期6例，Ⅲb期19例，Ⅳ期67例，中晚期（Ⅲb期及Ⅳ期）占87.8%（86/98）。Ⅳ期患者以双肺转移、骨转移及胸腔积液最为多见，其次为脑转移，发病时有两个或以上脏器转移的患者占80.6%（54/67）。

### 2.4 肿瘤治疗概况

5例患者确诊后放弃治疗，接受治疗率为95.1%（97/102）。97例接受治疗的患者中有6例接受手术治疗，术后肿瘤复发时间为2.5个月-42个月，中位复发时间为13.5个月。87例患者接受一线全身化疗，化疗周期数为1个-6个周期，客观有效率（objective response rate, ORR）为46.0%（40/87），疾病控制率（disease controled rate, DCR）为79.3%（69/87），肿瘤进展时间（time to progression, TTP）为1个月-15个月，中位TTP为5.0个月。

38例患者曾接受过分子靶向治疗，ORR为40.0%（14/35），DCR为65.7%（23/35），TTP为0.3个月-20.0个月，中位TTP为5.5个月。其中12例患者曾接受二次或多次TKI治疗，二次TKI治疗ORR为8.3%（1/12），DCR为66.7%（8/12），TTP为1.0个月-7.0个月，中位TTP为3.0个月。

10例患者曾接受*EGFR*基因突变检测（8例采用测序法，2例采用ARMS方法），有6例为阳性（阳性率60.0%，6/10），其中外显子19点突变3例，外显子21缺失突变3例，3例患者首次服用靶向治疗疗效为部分缓解（partial response, PR），余3例为稳定（stable disease, SD），ORR为50.0%（3/6），DCR为100.0%，TTP为2.0个月-9.0个月，中位TTP为2.8个月。

### 2.5 预后分析

采用患者定期住院复查、电话或短信随访的方式，自确诊时间起随访至患者死亡或截止日期2012年4月。中位随访时间为3年。截至2012年4月，11例患者仍存活。78例患者随访到死亡，其生存期（overall survival, OS）为4.0个月-61.0个月，中位生存期为10.5个月。统计97例接受治疗的患者自确诊至末次出院时间，84.5%（82/97）患者生存期 > 6个月，46.4%患者（45/97）生存期 > 12个月。其中7例患者确诊后仅接受过一线治疗，其OS分别为3.0个月和4.5个月；19例患者仅接受两线治疗，其中位OS为12.0个月；62例患者接受过三线或更多线的治疗，其中位OS为14.5个月。

## 3 讨论

相关文献^[6]^显示，肺癌在中老年患者的发病率趋于平稳甚至有下降趋势，而在青年患者中呈上升趋势。NSCLC占肺癌总发病率的80%左右，青年NSCLC值得临床关注。目前有关青年NSCLC的年龄界定尚未有统一的国际标准。近年来国内外文献多以发病年龄≤40岁作为青年NSCLC的年龄界定标准，部分文献将发病年龄＜45岁或＜50岁界定为青年NSCLC，因为30岁-45岁的青年人各系统处于相对平衡的状态，其手术耐受性、机体对肿瘤的免疫应答等与老年人有明显差异^[6, 7]^。目前所检索到的文献^[8]^显示最年轻的NSCLC为1例16岁的女性中分化鳞癌患者。本研究以绝大多数文献中采用的年龄≤40岁作为青年NSCLC的年龄界定标准，以便与国内外相关文献进行比较分析。

美国及欧洲各国40岁以下的青年NSCLC占全部患者的1.2%-6.0%^[3]^，据对SEER数据库31, 116例肺癌患者的回归性分析数据显示，年龄＜50岁的患者占总发病人数的9%，年龄＜40岁的青年患者占1%^[9]^；我国文献报道为3.1%-6.5%。从近年来的临床工作中发现，肺癌的发病年龄趋向于年轻化，具体数值尚要依靠大型的流行病学调查来证实。

文献^[10]^报道，青年NSCLC女性比例增加，且与吸烟的相关性减弱。本组资料显示，女性所占比例为42.4%，男女比例为1.36:1。不同国家文献报道的青年NSCLC男女比例不尽相同：一项来自美国的回顾性分析数据^[7]^显示其男女比例为1:1.12；波兰学者对757例年龄＜50岁的青年NSCLC患者回顾性分析结果显示：男女比例为2.88:1，此结果接近日本的2.5:1^[11]^；我国文献所报道的青年NSCLC中男女比例多在(1.2-2.3):1；与本组研究结果一致，这与文献报道的老年患者的(5-10):1有明显差别，即青年NSCLC患者中女性所占比例要明显高于老年患者。同时可知，欧美国家青年女性NSCLC发病率更高，这可能与其青年女性吸烟增加有关。本组患者中仅30.6%患者有吸烟史，且均为男性，仅1例女性患者有长期被动吸烟史。文献^[12]^报道西方国家90%以上的青年NSCLC患者有重度吸烟史，然而在部分亚洲国家的青年NSCLC患者中，重度吸烟者仅占40%-50%。可见吸烟与青年NSCLC的发生有一定相关性，但除此之外，仍有其它因素与NSCLC发生密切相关，这可能与遗传易感性、环境污染、饮食习惯改变及内分泌紊乱等有关。

近年来针对青年NSCLC遗传易感性的研究多有报道。文献^[13]^显示部分青年NSCLC患者存在GPX1198位点多态性，这与肺癌易感性有关，另外Lue198基因型携带者的肺癌发生率比其他类型高35%，除此之外，GPX1198多态性、EPHX1过表达亦增加了肺癌的易感性^[14]^。可见青年NSCLC发病与自身的基因分型有一定相关性，如能确认敏感性好的相关指标来对健康人群进行筛选，对易感人群进行定期监测，做到早期诊治，可能对改善青年NSCLC的预后有重要意义。

多数文献^[7]^显示NSCLC以腺癌多见，青年患者中腺癌所占比例高于老年NSCLC。本组资料中腺癌所占比例为77.5%，与文献报道结果一致。分析可能与以下因素有关：①过滤嘴的使用使得更细小的烟草致癌物达到肺的外周，亦即腺癌的好发部位；②与其它病理类型相比，腺癌更容易被诱发，较少的基因损伤即能诱导腺癌的发生；③青年NSCLC患者中女性所占比高于老年患者，女性易患肺癌，且更容易在较年轻及较少抽烟时患肺癌^[15]^；④老年患者鳞癌多于青年患者可能与呼吸道粘膜细胞受环境因素刺激和自身免疫调控缺陷，使发生鳞癌癌变的时间较长有关。

本组青年患者自出现首发症状至确诊的时间为2.0个月，与文献报道的1.5个月相近，而老年患者中位确诊时间为0.7个月，明显低于青年患者，Ayesha等^[6]^认为这可能与患者对疾病重视程度不够及临床医师对青年NSCLC的认识不足有关。文献报道Ⅰ期/Ⅱ期的早期患者在青年NSCLC中占4.8%-23%，老年患者为15%-54%，而本组资料显示青年NSCLC中Ⅰ期/Ⅱ期仅占6.1%，中晚期（Ⅲb期及Ⅳ期）占87.8%，这亦可能与肺部肿瘤内科收治的患者多为不能手术的中晚期患者有关。

本组资料中4.9%的患者放弃治疗，绝大多数患者确诊后接受了积极的抗肿瘤治疗。5例患者接受手术治疗，术后肿瘤复发时间为2.5个月-42个月，中位复发时间为13.5个月，可见青年NSCLC患者如能早发现早治疗仍能获得较长的生存期^[16]^。患者接受一线化疗的客观有效率为46.0%，疾病控制率为79.3%，中位TTP为5.0个月，可知青年NSCLC患者一线治疗疗效尚可，但总体TTP较短，提示青年肿瘤进展较为迅速，恶性程度相对较高，这可能与青年NSCLC分化差有关，这点在本组资料中亦得到验证。

38例患者曾接受过TKI分子靶向治疗，总的ORR为40%，DCR为65.7%，但中位TTP仅为5.5个月。6例*EGFR*基因突变阳性的患者其ORR为50%，疾控率为100%，但中位TTP仅为2.8个月，如此短的中位TTP时间笔者考虑可能与检测方法是否存在假阳性、总体例数过少有关，今后仍需大样本的临床资料来进一步明确。虽然如此，从以上数据中可推测青年NSCLC患者可从TKI治疗中获益，但可能总体肿瘤进展较快，肿瘤缓解时间短。12例先后接受过两次或多次TKI治疗的患者，其二次靶向治疗的DCR可达66.7%，中位TTP时间为3.0个月，笔者认为此部分患者二次TKI受益可能与其一线TKI治疗的疗效及治疗时间长有关，但总体例数较少，仍待大样本的研究来证实。

近年来*EML4*-*ALK*融合基因是研究的热点，针对此靶点的新型药物Crizotinib已在美国上市。2010年Zhang等^[17]^报道了一组103例NSCLC患者中*EML4*-*ALK*融合基因的频率，确定12例（11.6%）表达EML4-ALK，研究显示EML4-ALK融合与较年轻的发病年龄相关。我科近期筛选的*EML4*-*ALK*融合基因阳性的19例患者中，有8例为年龄＜40岁的青年NSCLC患者（42.1%），此部分患者在接受针对此基因的新型靶向药物Crizotinib治疗，这为部分青年NSCLC患者提供了新的治疗希望。

有关青年NSCLC患者的预后情况目前仍有分歧。有学者认为，与老年患者相比青年NSCLC进展极为迅速，预后差，这可能与其肿瘤分化程度低以及较高频率的血管受侵有关。但有的学者认为青年NSCLC患者体力状况优于老年患者，合并症较少，治疗的耐受性好，本组患者中有的青年NSCLC患者可接受至八线治疗，而老年患者极少能有八线治疗的机会。本组患者中位生存期为10.5个月，能接受多线治疗或分子靶向药物治疗可使患者生存获益。由于本组病例数有限，未进行进一步预后相关因素的分层分析。有关青年NSCLC的预后情况及其与老年NSCLC预后的差别仍待临床大样本的完整的随访资料来进一步明确及验证。

综上，青年肺癌有较老年患者不同的临床病理特征：①女性患者所占比例相对较高；②吸烟患者所占比例低；③体力状况好，合并症较少；④中位确诊时间较长；⑤腺癌所占比例较高；⑤以中晚期病例为主；⑥一线治疗疗效尚可，但缓解时间较短；⑦总体预后不佳。延误诊断、体力状况、临床分期及肿瘤分化程度均可能是影响预后的重要因素，另外如能从分子生物学及遗传学等角度分析其特征可能有助于对青年NSCLC有更深入的认识。
